# EHR perception and momentary well-being in oncology professionals

**DOI:** 10.1016/j.esmorw.2026.100754

**Published:** 2026-08-24

**Authors:** M.K. Wekenborg, C. Rominger, E.A.M. Michels, I.C. Wiest, L. Treiber, J. Eder, A.R. Schwerdtfeger, Jakob Nikolas Kather

**Affiliations:** 1 Else Kroener Fresenius Center for Digital Health, Faculty of Medicine and University Hospital Carl Gustav Carus, TUD Dresden University of Technology, Dresden, Germany; 2 Department of Health Psychology, Institute of Psychology, University of Graz, Graz, Austria; 3 Department of Medicine I, Faculty of Medicine and University Hospital Carl Gustav Carus, TUD Dresden University of Technology, Dresden, Germany; 4 Department of Medical Oncology, National Center for Tumor Diseases (NCT), Heidelberg University Hospital, Heidelberg, Germany; 5 Pathology and Data Analytics, Leeds Institute of Medical Research at St James’s, University of Leeds, Leeds, UK

**Keywords:** ambulatory assessment, electronic health record, heart rate variability, occupational well-being, oncology, system perception

## Abstract

**Background:**

Digital systems increasingly shape oncology workflows, yet their impact on professionals’ momentary well-being remains poorly understood. Existing evidence relies on retrospective self-reports and lacks real-time, multimodal data. We examined whether momentary perception of an oncology electronic health record (EHR), differentiated into instrumental quality (e.g. usability) and noninstrumental quality (e.g. visual esthetics), relates to oncology professionals’ concurrent psychophysiological well-being during routine clinical use.

**Materials and methods:**

In this multimodal ambulatory assessment study at a German tertiary university hospital, 22 oncology professionals provided 449 momentary self-report assessments during active EHR interaction. System perception, positive and negative affect, and acute stress were assessed at each prompt. Continuous electrocardiography was recorded in 18 participants (342 observations), from which heart rate variability (HRV) was derived for the 5-min window preceding each prompt. Multilevel regression models separated within- from between-person effects.

**Results:**

Both instrumental and noninstrumental quality independently predicted overall EHR system appraisal. The two dimensions showed distinct associations with momentary well-being: at the within-person level, higher instrumental quality was associated with more positive affect (β = 0.08, *P* = 0.012), less negative affect (β = −0.10, *P* = 0.014), and marginally lower acute stress (β = −0.06, *P* = 0.083). Higher noninstrumental quality was linked to higher HRV (β = 0.12, *P* = 0.002).

**Conclusions:**

Momentary EHR system perception is associated with multiple facets of psychophysiological well-being during routine oncology workflows. As sustainable cancer care depends on the well-being of oncology professionals, clinical software emerges as a design-accessible correlate relevant to system sustainability.

## Highlights

• First real-time multimodal study linking EHR perception to well-being in oncology professionals.
• Multiple facets of EHR system perception are linked to oncology professionals’ momentary well-being.
• Perceived EHR quality relates to affect and HRV in distinct patterns.
• Results point to EHR system perception as a designable factor relevant for occupational well-being.

## Introduction

There is little doubt that advances in artificial intelligence (AI) will fundamentally transform the digital infrastructure of oncology within a remarkably short time.^1^ This expansion seems inevitable, and it carries enormous potential, not only for improving patient outcomes and operational efficiency but also for the well-being of those who deliver cancer care. Oncology professionals, including physicians, nurses, and clinical documentation specialists, are among the occupational groups most frequently affected by chronic work-related stress and its serious health consequences worldwide.^2^^,^^3^ How professionals perceive the digital systems they interact with throughout their workday, and how these perceptions relate to their momentary well-being, may represent a design-accessible correlate of this burden. These processes are commonly conceptualized as user experience and can be directly shaped through system design. Designing digital systems that actively support rather than undermine their momentary well-being is thus not only an opportunity afforded by this technological transition, but it is a clinical and organizational imperative. Yet, the current reality falls short of this potential. Digitalization at work is frequently experienced as an additional burden rather than a source of well-being,^4^ a pattern particularly pronounced in healthcare.^5^ In oncology, electronic health records (EHRs) exemplify this tension. Despite structuring nearly every aspect of clinical practice, their use has been associated with increased burden^6^^,^^7^ and higher stress and burnout symptoms^8^ among oncology professionals. This is a serious concern for reasons that extend well beyond the individual health of oncology professionals. Impaired well-being has been associated with reduced care quality and compromised workforce sustainability, including intentions to reduce clinical hours and leave practice.^9^^,^^10^ At the same time, systems that burden rather than support their users risk reduced acceptance and suboptimal adoption, undermining the success of digitalization itself (for review, see Nastjuk et al.^11^).

The few existing studies on EHR use and well-being in oncology are important, as they highlight the need to improve digital system design for a health-promoting digitalization of oncology. However, they almost exclusively rely on retrospective self-reports, typically administered at considerable temporal distance from actual system use. This approach is vulnerable to recall bias and misses moment-to-moment fluctuations. Moreover, it is limited to subjective, self-reported experience, although well-being is a multifaceted construct that encompasses both psychological and physiological processes, which do not necessarily converge.^12^ Among physiological indicators, heart rate variability (HRV), reflecting variations in interbeat intervals,^13^^,^^14^ is particularly relevant for capturing health-related momentary physiological changes during real-world system use. Its widespread use in ambulatory studies stems from being both reliably associated with health and well-being^13^^,^^15^ and, due to advances in wearable technology, being reliably and scalably measurable in everyday settings.

Laboratory-based approaches have begun to examine how software characteristics relate to individuals’ momentary experience using psychophysiological methods (for review, see Da Silveira et al.^16^), including HRV.^17^ However, the specific association between system perception dimensions and well-being has been largely neglected. Moreover, existing work has not extended to real-world clinical settings, where multitasking, frequent interruptions, and the emotional weight of cancer care create conditions that fundamentally differ from controlled environments.

As a result, we know little about how specific, modifiable qualities of oncology EHR systems are associated with the momentary well-being of oncology professionals during routine use. This gap is consequential: without real-time, multimodal evidence linking specific system properties to momentary well-being, efforts to design digital tools that support rather than burden oncology professionals remain largely without empirical foundation. To address this, we apply for the first time in oncology a real-time multimodal assessment strategy during active EHR interaction, focusing on theoretically and empirically established dimensions of system perception, differentiated into instrumental qualities (e.g. usability) and noninstrumental qualities (e.g. visual esthetics) and test whether these are associated with oncology professionals’ psychophysiological momentary well-being during routine use, namely positive and negative affect, acute stress, and HRV.

## Materials and methods

### Study design and participants

**Figure 1:**
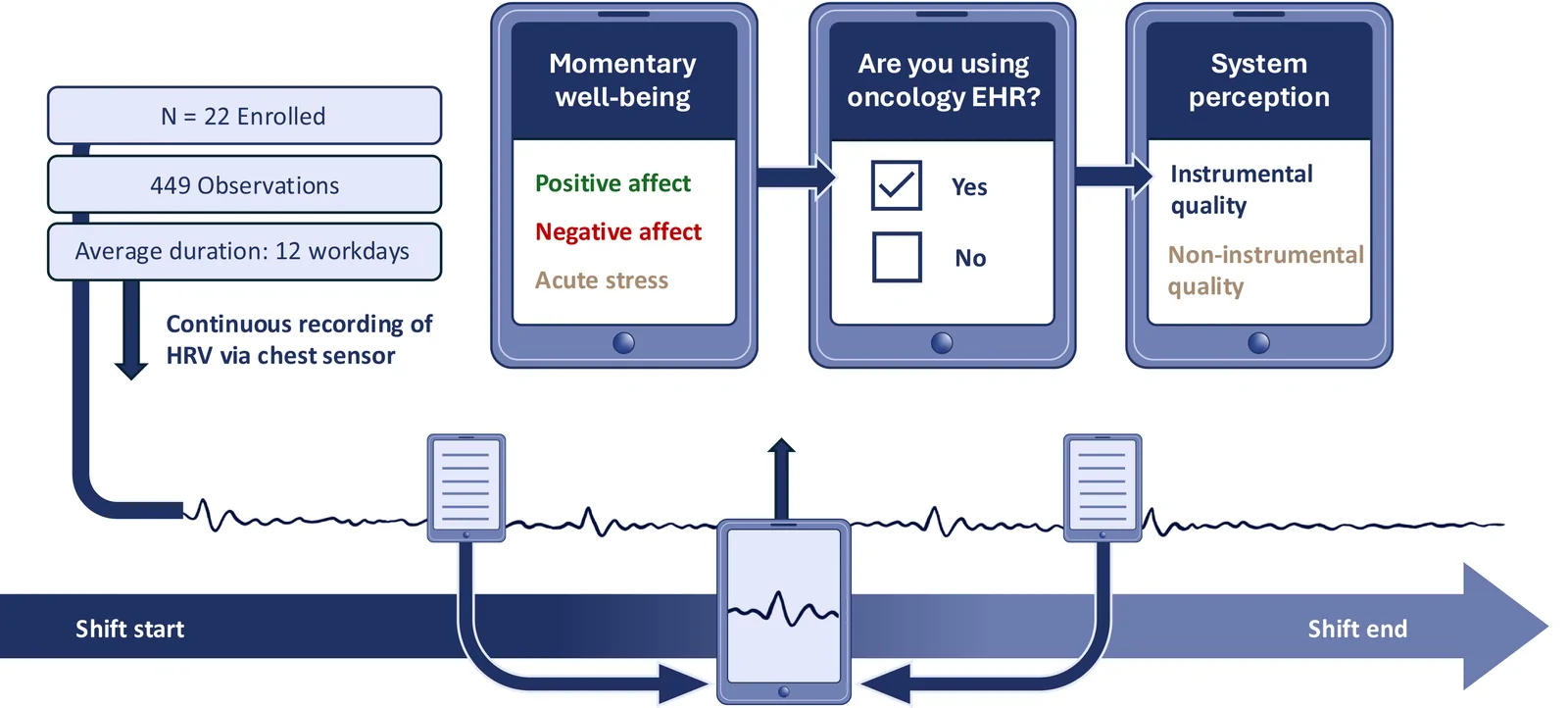
**Real-time multimodal assessment of clinician experience during EHR use in oncology.** After baseline assessment, participants were equipped with a wearable ECG device for continuous recording during routine work shifts. Throughout the assessment period, up to eight prompts per shift were delivered at randomized intervals. At each prompt, participants reported their momentary affect and acute stress and indicated whether they had interacted with the EHR in the preceding 5 min. Prompts confirmed as occurring during active EHR use triggered additional assessments of product perception and system appraisal. Concurrently, HRV was derived from the 5-min window preceding each prompt. Data collection continued until ∼50 prompts per participant were obtained. ECG, electrocardiography; EHR, electronic health record; HRV, heart rate variability.

This was a multimodal ambulatory observational study conducted at a tertiary university hospital in Germany (Figure 1). Eligible participants were healthcare professionals routinely involved in oncological documentation. Inclusion criteria were (i) current employment at the participating hospital, (ii) age ≥18 years, (iii) regular use of the oncology EHR as part of routine clinical workflow, and (iv) absence of any cardiovascular condition or medication with known effects on cardiovascular function. Of 25 professionals enrolled through internal hospital communication channels, momentary self-report data from 22 were available for the present analyses (449 momentary observations during active EHR interaction). For analyses involving HRV, 18 participants and 342 observations were available [exclusion due to medical contraindication for electrocardiography (ECG) wear (*n* = 1), insufficient ECG signal quality (*n* = 1), or incomplete concurrent self-report data (*n* = 2)]. No data were imputed. The study was approved by the ethics committee of TU Dresden, Germany [approval date: 4 March 2025; approval ID: BO ff (Mono)-EK-40012025] and conducted in accordance with the Declaration of Helsinki. All participants provided written informed consent before enrollment.

### Procedures

One week before onboarding, participants received an email link to a baseline questionnaire hosted on LimeSurvey (LimeSurvey GmbH, Hamburg, Germany), capturing informed consent, sociodemographic characteristics, and health-related variables. Onboarding took place individually at each participant’s workplace and included oral and written instructions on the ambulatory protocol. Participants were equipped with an EcgMove4 chest sensor (movisens GmbH, Karlsruhe, Germany), fitted on a custom chest strap for continuous ECG recording from shift start to shift end throughout the assessment period. Momentary self-reports were administered via the movisensXS application (movisens GmbH), installed on the participant’s personal smartphone or, alternatively, on a study device provided on request. Each evening, participants reported their next-day shift schedule through a brief evening survey, enabling individualized prompt delivery, alongside additional daily measures not considered in the present analyses. During working hours, up to eight prompts were issued at randomized intervals, separated by a minimum of 30 min, with the actual number depending on shift length. At each prompt, participants rated their affect and acute stress over the preceding 5 min and indicated whether they had been interacting with the oncology EHR during that interval. Only prompts confirmed as occurring during active EHR use triggered the full assessment of product perception and system appraisal. Data collection continued on an individual basis until 50 prompts were obtained per participant, after which the ECG sensor was retrieved at the workplace. As compensation, participants received an individualized feedback report summarizing their physiological stress profile during the assessment period.

### Measures

#### Outcomes

Four indicators of momentary psychophysiological well-being constituted the central study endpoints: positive affect, negative affect, and acute stress (self-reported), as well as HRV (physiological). Two indicators of overall EHR system appraisal, consequences of use (intention to use and product loyalty) and overall evaluation (global attractiveness), served as additional outcomes to empirically validate the real-time product perception assessment under routine clinical conditions.

#### Self-report measures

All self-report items used five-point Likert response scales (1 = not at all to 5 = extremely). Momentary perception of the oncology EHR was operationalized using 10 items adapted from the modular evaluation of key Components of User Experience questionnaire (meCUE 2.0, Technische Universität Berlin, Berlin, Germany^18^), covering instrumental quality (usefulness and usability) and noninstrumental quality (visual esthetics, status, and commitment). The meCUE 2.0 additionally provided the two system appraisal indicators described earlier. Momentary affect was assessed using eight items (four per dimension) from the validated German short version of the Positive and Negative Affect Schedule (PANAS; Bianka Breyer and Matthias Bluemke, GESIS - Leibniz Insitute for the Social Sciences, Mannheim, Germany^19^), selected for its psychometric robustness in ambulatory research contexts.^20^ Acute stress was captured with a single item (‘Were you stressed in the last 5 minutes?’), previously applied in ambulatory research.^21^

#### HRV

Continuous ECG was recorded at 1024 Hz via the EcgMove4 sensor in a modified Einthoven II configuration. HRV was quantified as the root mean square of successive differences between adjacent R-R intervals (RMSSD), computed for the 5-min window immediately preceding each prompt using DataAnalyzer software (movisens GmbH). RMSSD is a well-established short-term index of vagally mediated HRV, suited to ambulatory designs for its efficiency, robustness to respiratory variation, and prevalence in prior ecological momentary assessment (EMA) research.^21^, ^22^, ^23^, ^24^, ^25^ RMSSD values were natural log-transformed to correct for skewness. Concurrent physical activity, expressed as metabolic equivalents (MET) derived from the device’s built-in accelerometer, was extracted for the same 5-min window and included as a covariate in HRV models to adjust for metabolic autonomic variation.

### Statistical analyses

All analyses were carried out in R^26^ using the lme4 (v. 1.1-38; Douglas Bates, University of Wisconsin-Madison, Madison, WI^27^), lmerTest (v. 3.2-0; Alexandra Kuznetsova, Technical University of Denmark, Kongens Lyngby, Denmark^28^), performance (v. 0.16.0; Daniel Lüdecke, University Medical Center Hamburg-Eppendorf, Hamburg, Germany^29^), and effectsize (v. 1.0.1; Mattan S. Ben-Shachar, Ben-Gurion University of the Negev, Beer-Sheva, Israel^30^) packages. Figures were created using the ggplot2 package (v. 4.0.2; Hadley Wickham, Posit, PBC, Boston, MA^31^). The nested data structure (multiple assessments within participants) was modeled using multilevel regression. All time-varying predictors were decomposed into a person mean and a person-mean-centered deviation score to separate between- from within-person effects. Model structure was determined through a systematic comparison of random-intercept and random-slope specifications.

For each outcome, random-slope specifications were added individually to a random-intercept baseline, checked for singularity, and the nonsingular candidates compared with the baseline using likelihood ratio tests, Akaike information criterion, and Bayesian information criterion (BIC) (maximum likelihood estimation). In case of divergent indices, the BIC was prioritized. Final models were re-estimated via restricted maximum likelihood for parameter reporting (Supplementary Table S1, available at https://doi.org/10.1016/j.esmorw.2026.100754), with coefficients standardized using the effectsize package (refit method).

Analyses proceeded in two steps. As a prerequisite, we verified that the two product perception dimensions captured during real-time assessment were meaningfully linked to overall EHR system appraisal. To this end, instrumental and noninstrumental product perception were entered simultaneously as predictors of two appraisal indicators in separate models: consequences of use and overall evaluation. As sensitivity analyses, the unique contribution of each product perception dimension was assessed by comparing the full model against reduced models omitting one dimension at a time. The central analysis then tested whether the same two dimensions predicted momentary psychophysiological well-being. Four outcomes were modeled separately: positive affect, negative affect, acute stress, and HRV. The HRV model additionally included MET as a covariate at both the within- and between-person level. A two-sided *P* value of <0.05 was considered statistically significant.

## Results

### Participant characteristics

**Figure 2:**
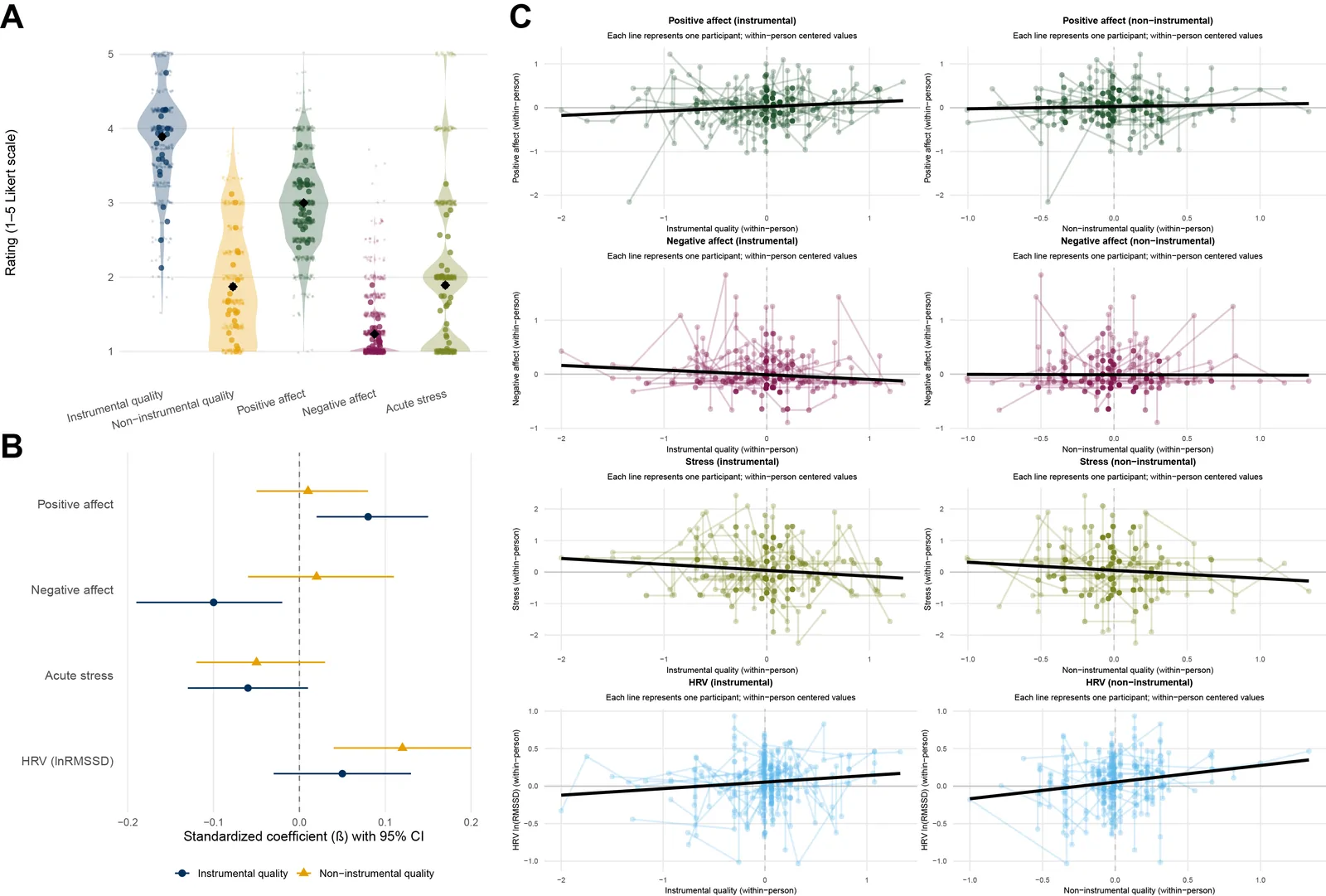
**Momentary measures and within-person associations between product perception and well-being during routine EHR interaction (*N* = 22, 449 observations; HRV: *N* = 18, 342 observations).** Well-being was operationalized as positive affect, negative affect, acute stress, and HRV. **(A)** Distributions of momentary product perception and well-being indicators on a five-point Likert scale: violins show overall distributions, small points individual observations, larger points participant means, and black diamonds grand means ± standard error. **(B)** Standardized within-person coefficients (β, 95% CI) from multilevel models predicting each well-being outcome from instrumental (blue) and noninstrumental quality (orange). Dashed line marks zero. **(C)** Within-person scatter plots for each predictor–outcome combination; thin lines connect repeated observations within participants, bold lines show overall within-person regressions. All variables person-mean centered. CI, confidence interval; EHR, electronic health record; HRV, heart rate variability.

A total of 22 oncology professionals contributed evaluable momentary self-report data during active EHR interaction (17 females; mean age 41.0 years, standard deviation = 8.5), including medical documentation specialists (*n* = 13) and study coordinators (*n* = 3). HRV analyses were based on a subsample of 18 participants with 342 concurrent observations. Intraclass correlations indicated that between-person differences accounted for a substantial proportion of variance across all outcomes (intraclass correlation coefficients between 0.27 and 0.74; Supplementary Table S2, available at https://doi.org/10.1016/j.esmorw.2026.100754). Descriptive statistics of all psychophysiological momentary measures are shown in Figure 2A.

### Product perception and EHR system appraisal

Before testing associations with momentary well-being, we verified that the two product perception dimensions captured in real-time, instrumental quality (usability and usefulness) and noninstrumental quality (visual esthetics, status, and commitment), were in fact associated with overall EHR appraisal, empirically anchoring their theoretically assumed role as central determinants of oncology professionals’ global system evaluation. The two indicators of global EHR evaluation (consequences of use and overall evaluation of the system) were examined in separate models.

For consequences of use, both instrumental (β = 0.13, *P* < 0.001) and noninstrumental quality (β = 0.12, *P* < 0.001) were significantly associated with the outcome at the within-person level. At the between-person level, only noninstrumental quality showed a significant association (β = 0.61, *P* = 0.002). Sensitivity analyses confirmed that both product perception dimensions contributed independently (both *P* < 0.001; Supplementary Table S1, available at https://doi.org/10.1016/j.esmorw.2026.100754). For overall evaluation of the system, both instrumental (β = 0.33, *P* < 0.001) and noninstrumental quality (β = 0.09, *P* = 0.001) were associated with the outcome at the within-person level, whereas at the between-person level, only instrumental quality reached significance (β = 0.47, *P* < 0.001). Both dimensions again contributed independently (both *P* ≤ 0.002; Supplementary Table S1, available at https://doi.org/10.1016/j.esmorw.2026.100754). Across both appraisal outcomes, marginal *R*^2^ values ranged from 0.37 to 0.46, indicating that product perception captured a substantial portion of the variance in overall EHR appraisal.

Together, these data show that both product perception dimensions were independently associated with overall EHR evaluation, supporting the validity of their real-time assessment even under routine clinical conditions.

### Product perception and clinicians’ momentary well-being

We next examined whether instrumental and noninstrumental quality were associated with four indicators of momentary well-being during active EHR interaction: positive affect, negative affect, acute stress, and HRV. Within-person coefficients are summarized in Figure 2B and illustrated for each predictor–outcome combination in Figure 2C. Full results including between-person associations are reported in Table 1.

**Table 1:** Standardized within- and between-person associations of product perception with momentary well-being during active EHR interaction

		Self-reported indicators			Physiological indicator
Predictor	Level	Positive affect	Negative affect	Acute stress	HRV (lnRMSSD)
Product perception					
Instrumental quality	Within	0.08 [0.02, 0.15] *P* = **0.012**	−0.10 [−0.19, −0.02] *P* = **0.014**	−0.06 [−0.13, 0.01] *P* = 0.083	0.05 [−0.03, 0.13] *P* = 0.212
Noninstrumental quality	Within	0.01 [−0.05, 0.08] *P* = 0.718	0.02 [−0.06, 0.11] *P* = 0.589	−0.05 [−0.12, 0.03] *P* = 0.212	0.12 [0.04, 0.20] *P* = **0.002**
Instrumental quality	Between	0.02 [−0.30, 0.35] *P* = 0.893	0.05 [−0.20, 0.29] *P* = 0.709	0.04 [−0.22, 0.30] *P* = 0.781	0.28 [0.02, 0.55] *P* = **0.048**
Noninstrumental quality	Between	0.21 [−0.20, 0.62] *P* = 0.323	−0.10 [−0.39, 0.19] *P* = 0.522	−0.26 [−0.58, 0.06] *P* = 0.128	0.13 [−0.22, 0.49] *P* = 0.475
Covariate					
Physical activity (MET)	Within	–	–	–	−0.04 [−0.12, 0.04] *P* = 0.294
Physical activity (MET)	Between	–	–	–	0.14 [−0.21, 0.49] *P* = 0.451

Standardized coefficients (β) with 95% CIs from multilevel models. Within = person-mean-centered (within-person fluctuations); between = person mean (between-person differences). The HRV model included physical activity (MET) as a covariate at both levels. Bold *P* values indicate statistical significance (*P* < 0.05). Self-reported indicators: *N* = 22 participants, 449 observations. HRV: *N* = 18 participants, 342 observations.

CI, confidence interval; EHR, electronic health record; HRV, heart rate variability; lnRMSSD, natural logarithm of the root mean square of successive differences between adjacent R-R intervals; MET, metabolic equivalent.

With respect to the self-reported psychological indicators, instrumental quality was significantly associated at the within-person level with positive affect (β = 0.08, *P* = 0.012) and negative affect (β = −0.10, *P* = 0.014), indicating that moments of higher perceived usability and usefulness coincided with more positive and less negative affective states. A marginally significant association was observed with acute stress (β = −0.06, *P* = 0.083), indicating that moments of higher perceived usability and usefulness tended to coincide with lower acute stress. Noninstrumental quality was not associated with any of these affective indicators at the within-person level.

With respect to the physiological indicator, with physical activity (MET) included as a covariate at both levels, noninstrumental quality was significantly associated with HRV at the within-person level (β = 0.12, *P* = 0.002), indicating that moments of higher perceived esthetic and design quality coincided with higher HRV. At the between-person level, instrumental quality significantly predicted HRV (β = 0.28, *P* = 0.048), suggesting that professionals who generally perceived the system as more usable exhibited higher overall HRV.

These data show that product perception during active EHR interaction is associated with multiple indicators of momentary psychophysiological well-being.

## Discussion

In this study, we found that momentary perception of the oncology EHR, assessed directly during active clinical use, was associated with oncology professionals’ concurrent psychophysiological well-being. Both instrumental quality (usability and usefulness) and noninstrumental quality (visual esthetics, status, and commitment) independently predicted overall EHR system evaluation, empirically anchoring their role as central determinants of system appraisal. Crucially, the two dimensions showed distinct associations with momentary well-being during active use: higher perceived instrumental quality was linked to more favorable self-reported affective states, whereas higher perceived noninstrumental quality was linked to higher HRV. These findings indicate that system perception is an experientially meaningful and psychophysiologically relevant property of oncology EHR systems under real-world clinical conditions.

To our knowledge, this is the first study to link modifiable perception dimensions of an oncology EHR to concurrent psychological and physiological well-being during routine clinical use. Crucially, the dimensions examined here are properties of the system itself and are, in principle, accessible to design optimization, unlike many other sources of occupational strain in oncology that are inherent to the nature of cancer care.

Existing evidence in this field has relied on retrospective, self-report-based, and global assessments,^8^ which cannot capture how system properties shape well-being within individuals as they unfold during actual interaction. This type of fine-grained real-time multimodal evidence is, however, essential for understanding which specific EHR system properties matter for oncology professionals in the particular work context in which they operate and for targeting these properties in system optimization. Such efforts appear increasingly urgent given the substantial mental health burden among oncology professionals.^2^^,^^3^

Our finding that both instrumental and noninstrumental product perception contributed independently to overall EHR appraisal aligns with established frameworks that conceptualize these dimensions as jointly constitutive of system evaluation.^17^^,^^32^ Extending this evidence, we show that this dual structure holds even when the system is evaluated under the demands of oncology workflows, which differ fundamentally from the controlled laboratory settings in which EHR system properties are usually evaluated. This convergence provides initial evidence for the validity of our multimodal assessment approach in real-world clinical contexts.

Importantly, psychological and physiological indicators of well-being showed distinct association patterns with system perception: instrumental quality was linked to more favorable affective states, whereas noninstrumental quality was associated with higher HRV. Although context-dependent, higher HRV is generally considered favorable, as it reflects enhanced cardiac parasympathetic activity and associated metabolic resource conservation,^13^^,^^33^, and has been associated with greater self-regulatory capacity and reduced stress.^15^ The within-person effect sizes are modest, as expected for momentary fluctuations during routine clinical activity. Their relevance lies less in any single association than in their cumulative nature: because EHR interaction is among the most frequent activities of the clinical day, small momentary effects may aggregate over numerous daily exposures. We nonetheless interpret these associations as signals warranting replication rather than as established clinically meaningful effects. The limited existing evidence on the link between system perception dimensions and psychophysiological responses is restricted to laboratory settings and suggests a different pattern: poor instrumental quality has been linked to both affective and physiological responses (e.g. electrodermal activity), whereas noninstrumental quality has shown effects on affect without clear effects on physiological measures.^17^ In addition, outside the context of digital systems, laboratory studies have shown that visual stimuli can elicit measurable autonomic responses that are not necessarily mirrored in explicit affect reports.^34^ These inconsistent findings highlight the need for further research, particularly in real-world contexts where associations may differ from those observed under laboratory conditions.

At the same time, the current evidence underscores the added value of incorporating physiological markers beyond self-report when assessing well-being in the context of digital system use. With the ongoing development of wearable devices capable of continuously deriving HRV, this opens a scalable and minimally intrusive avenue for system evaluation under routine clinical conditions.

Several limitations should be considered. The sample was relatively small; yet, the emergence of consistent, theoretically coherent associations across both psychological and physiological indicators, despite this sample size, underscores the relevance of the observed effects. Still, sensitivity to detect more subtle associations may have been limited, and replication in larger samples is warranted. The study focused on a single EHR system in one clinical context, which ensures internal consistency but limits transferability. External validity is constrained in several respects. Participation was voluntary and drawn from a single tertiary center, so professionals with stronger EHR views, greater digital affinity, or greater willingness to wear an ECG device may be overrepresented. The sample also consisted predominantly of documentation specialists and study coordinators. The associations should therefore be read as evidence of feasibility and of a within-person signal rather than as estimates representative of the broader oncology workforce, and replication across professions and centers is warranted. The exposure and three of the four outcomes were obtained by concurrent self-report, raising the possibility of common-method variance, affect-congruent responding, and response-consistency bias. This does not extend to the HRV findings: HRV is an objective, method-independent index, so the association between noninstrumental quality and HRV cannot be attributed to shared-method artifacts. The convergence of self-reported and physiological indicators across distinct modalities mitigates, although does not eliminate, this concern. The HRV models adjusted for physical activity (MET), a physiological prerequisite for interpreting vagally mediated HRV, but did not model other time-varying contextual factors such as workload, patient complexity, interruptions, time pressure, setting, time of day, fatigue, or day of week, which may confound the observed associations and should be measured directly in future work. Stable person-level characteristics (age, sex, and profession) do not bias the person-mean-centered within-person estimates; given the sample size, we deliberately limited between-person covariates to avoid overfitting at the person level. Finally, because system perception and momentary well-being were measured concurrently, the temporal order of these associations cannot be determined, and reverse causation is equally plausible: professionals in a more positive affective or lower-stress state may perceive the system more favorably. Our findings therefore identify system perception as a design-accessible correlate of momentary well-being; they do not establish that modifying software design will, by itself, improve well-being. Experimental designs with targeted system modifications are required to test this directionality. In general, future research should replicate these findings across multiple systems and contexts, and employ experimental designs, including targeted system modifications, to clarify causal pathways and extend this approach to emerging digital tools such as AI-based systems. Oncology workflows increasingly incorporate AI-assisted decision support and large language models within EHR ecosystems. Because the perception dimensions examined here are generic properties of human–system interaction rather than features specific to documentation, the observed associations may plausibly extend to AI-enabled interfaces and clinical decision support, where real-time multimodal assessment of the kind demonstrated here could evaluate their experiential and physiological impact during routine use. Whether these associations generalize is an empirical question for future research.

### Conclusion

In this study, momentary perceptions of an oncology EHR system were empirically linked to concurrent psychophysiological well-being during routine clinical use, with instrumental and noninstrumental qualities relating to distinct facets of well-being. These findings identify system perception as a design-accessible correlate of well-being during EHR interaction and show that scalable real-time multimodal assessment can capture these dynamics in everyday clinical workflows. As the digital infrastructure of oncology expands, attending to the experiential quality of clinical software may be an important element to sustainable cancer care delivery.
