# Supplementary material for: EHR perception and momentary well-being in oncology professionals
Source: ESMO Real World Data Digit Oncol. 2026 Aug 24;13:100754. doi: 10.1016/j.esmorw.2026.100754 (PMC13542604; doi:10.1016/j.esmorw.2026.100754)
Supplement: Supplementary file 1 [file mmc1.docx]

# Supplements

### List of Supplementary Tables

Table S1. Full multilevel model results for the prediction of EHR system appraisal indicators by instrumental and non-instrumental quality.

Table S2. Intraclass correlations (ICCs) for all outcome variables.

**Table S1.** Full multilevel model results for the prediction of EHR system appraisal indicators by instrumental and non-instrumental quality.

| Model | Predictor |  | β [CI] / χ²(df) | SE | df | t | P |
| --- | --- | --- | --- | --- | --- | --- | --- |
| **Consequences of use (RI)** | **(Intercept)** |  | -0.05 [-0.34, 0.25] | 0.796 | 20.38 | 1.51 | .146 |
|  | Non-instrumental quality | Between | 0.61 [0.29, 0.93] | 0.218 | 17.94 | 3.74 | .002 |
|  | Non-instrumental quality | Within | 0.12 [0.07, 0.16] | 0.071 | 425.19 | 5.31 | < .001 |
|  | Instrumental quality | Between | -0.11 [-0.36, 0.14] | 0.221 | 20.30 | -0.86 | .399 |
|  | Instrumental quality | Within | 0.13 [0.09, 0.18] | 0.046 | 425.19 | 6.12 | < .001 |
| R²m = 0.37 |  |  |  |  |  |  |  |
| R²c = 0.80 |  |  |  |  |  |  |  |
| **Overall evaluation (RS: instrumental)** | **(Intercept)** |  | -0.06 [-0.29, 0.18] | 0.624 | 19.77 | -0.25 | .809 |
|  | Instrumental quality | Between | 0.47 [0.27, 0.67] | 0.174 | 20.05 | 4.57 | < .001 |
|  | Instrumental quality | Within | 0.33 [0.24, 0.43] | 0.100 | 14.96 | 6.90 | < .001 |
|  | Non-instrumental quality | Between | 0.20 [-0.04, 0.44] | 0.163 | 15.71 | 1.61 | .128 |
|  | Non-instrumental quality | Within | 0.09 [0.04, 0.15] | 0.092 | 414.63 | 3.23 | .001 |
| R²m = 0.46 |  |  |  |  |  |  |  |
| R²c = 0.72 |  |  |  |  |  |  |  |
|  |  |  |  |  |  |  |  |
| Nested model comparisons (Consequences) | Drop instrumental |  | χ²(2) = 36.80 |  |  |  | < .001 |
|  | Drop non-instrumental |  | χ²(2) = 39.50 |  |  |  | < .001 |
|  |  |  |  |  |  |  |  |
| Nested model comparisons (Overall) | Drop instrumental |  | χ²(2) = 36.56 |  |  |  | < .001 |
|  | Drop non-instrumental |  | χ²(2) = 13.00 |  |  |  | .002 |

***Note.*** *Multilevel linear models estimated using restricted maximum likelihood (REML) for parameter reporting. “RI” denotes random-intercept models; “RS” denotes models including a random slope for the within-person instrumental predictor. Between-person effects are represented by group means, and within-person effects by group-mean centered deviations. Degrees of freedom (df) were estimated using the Satterthwaite approximation. R²m and R²c denote marginal and conditional R², respectively. Nested model comparisons were conducted using likelihood ratio tests based on maximum likelihood (ML) estimation. χ² values indicate differences relative to the full model.*

**Table S2.** Intraclass correlations (ICCs) for all outcome variables.

| Variable | ICC |
| --- | --- |
| Consequences of use | 0.736 |
| Overall evaluation | 0.522 |
| Positive affect | 0.596 |
| Negative affect | 0.267 |
| Acute stress | 0.432 |
| HRV (lnRMSSD) | 0.511 |

***Note****. Intraclass correlations (ICC) derived from intercept-only multilevel models with random intercepts for participants. ICC values represent the proportion of total variance attributable to between-person differences. HRV = heart rate variability; lnRMSSD = natural log-transformed root mean square of successive difference.*
